# Ion Chromatography Based Urine Amino Acid Profiling Applied for Diagnosis of Gastric Cancer

**DOI:** 10.1155/2012/474907

**Published:** 2012-07-25

**Authors:** Jing Fan, Jing Hong, Jun-Duo Hu, Jin-Lian Chen

**Affiliations:** ^1^Department of Gastroenterology, Shanghai Sixth People's Hospital, Shanghai Jiao Tong University, Shanghai 200233, China; ^2^Medical College, Soochow University, Suzhou, Jiangsu 215213, China; ^3^Department of Gastroenterology, Shanghai East Hospital, Tongji University, Shanghai 200120, China

## Abstract

*Aim*. Amino acid metabolism in cancer patients differs from that in healthy people. In the study, we performed urine-free amino acid profile of gastric cancer at different stages and health subjects to explore potential biomarkers for diagnosing or screening gastric cancer. *Methods*. Forty three urine samples were collected from inpatients and healthy adults who were divided into 4 groups. Healthy adults were in group A (*n* = 15), early gastric cancer inpatients in group B (*n* = 7), and advanced gastric cancer inpatients in group C (*n* = 16); in addition, two healthy adults and three advanced gastric cancer inpatients were in group D (*n* = 5) to test models. We performed urine amino acids profile of each group by applying ion chromatography (IC) technique and analyzed urine amino acids according to chromatogram of amino acids standard solution. The data we obtained were processed with statistical analysis. A diagnostic model was constructed to discriminate gastric cancer from healthy individuals and another diagnostic model for clinical staging by principal component analysis. Differentiation performance was validated by the area under the curve (AUC) of receiver-operating characteristic (ROC) curves. *Results*. The urine-free amino acid profile of gastric cancer patients changed to a certain degree compared with that of healthy adults. Compared with healthy adult group, the levels of valine, isoleucine, and leucine increased (*P* < 0.05), but the levels of histidine and methionine decreased (*P* < 0.05), and aspartate decreased significantly (*P* < 0.01). The urine amino acid profile was also different between early and advanced gastric cancer groups. Compared with early gastric cancer, the levels of isoleucine and valine decreased in advanced gastric cancer (*P* < 0.05). A diagnosis model constructed for gastric cancer with AUC value of 0.936 tested by group D showed that 4 samples could coincide with it. Another diagnosis model for clinical staging with an AUC value of 0.902 tested by 3 advanced gastric cancer inpatients of group D showed that all could coincide with the model. *Conclusions*. The noticeable differences of urine-free amino acid profiles between gastric cancer patients and healthy adults indicate that such amino acids as valine, isoleucine, leucine, methionine, histidine and aspartate are important metabolites in cell multiplication and gene expression during tumor growth and metastatic process. The study suggests that urine-free amino acid profiling is of potential value for screening or diagnosing gastric cancer.

## 1. Introduction

Gastric cancer is one of the most common malignancies and the second cause of cancer-associated death worldwide [1, 2]. The early diagnosis is very difficult because there are no specific symptoms at an early stage of gastric cancer, and early gastric cancer is typically small [3, 4]. Clinically, most gastric cancers were identified when they were at an advanced stage. Advanced gastric cancer which has a high mortality for its local and distant metastases does a great harm to human's health [4–8]. Up to now, we are not able to carry out any effective causal prophylaxis because the etiopathogenesis of gastric cancer is not defined [9, 10]; therefore, early diagnosis or screening is especially important to gastric cancer. Although endoscopy combining biopsy is a fairly mature method now, the rate of diagnosis is still relying on the experience of endoscopists and gastrointestinal pathologist [11, 12]. The serologic tests for gastric cancer such as CEA have little diagnosis value for their lower specificity and sensibility [13–15].

Amino acids in human body include exogenous and endogenous amino acids. They are distributed allover the body to participate in metabolism, called amino acid metabolic pool. Endogenous amino acids which are produced from protein degradation in tissue can participate in varied physiological adjustments, such as gene expression, cell multiplication, and inflammatory reaction. The fast speed of cell multiplication and prosperity metabolism is characteristic of malignancy [16]. So malignant cells need a large number of amino acids from amino acid metabolic pool to synthesize protein and nucleic acids. An abnormal plasma-free amino acid (PFAA) profile might be presented for the total reflection of cancer-induced protein metabolism in tumors, skeletal muscle, and liver in cancer patients. Some studies indicated that amino acid metabolism is not the same in different types of malignant tumors. Kubota et al. [17] studied PFAA concentrations in 58 cancer patients, including 22 breast cancer, 24 gastrointestinal cancer, and 12 head and neck cancer. The results showed that the seven amino acids (glutamine, threonine, histidine, cysteine, alanine, arginine, and ornithine) had a close link with specific cancers, indicating that PFAA profiles correlate with the organ-site origin among the three different malignant tumors. Reduction of gluconeogenic amino acids has been observed in early tumor growth in an animal study [18]. This reduction occurred as early as 6 days after tumor cell inoculation, when the tumor was not detectable. The staging of cancer characterized by tumor size, depth of invasion, and metastasis is considered to be related with the PFAA profile [19].

In recent years, metabonomics as a branch of systems biology has developed rapidly. Now,  it has been established as an extremely powerful analytical tool and hence found successful applications in many research areas including molecular pathology and physiology, drug efficacy and toxicity, gene modifications and functional genomics, environmental sciences, and disease diagnoses [20–26]. In oncology, metabonomics can apply various advanced techniques such as nuclear magnetic resonance (NMR), high-performance liquid chromatography/mass spectrometry (HPLC/MS, and LC/MS/MS), Fourier-transform infrared (FT/IR) spectroscopy, and gas chromatography/mass spectrometry (GC/MS) to detect and measure low-molecular-weight metabolites in animal and human body fluid (blood, urine, etc.) [27–32]. Metabonomics combining chemometrics can reveal metabolic changes in malignant tumors and show powerful values in clinical study.

 Ion chromatography (IC) has been proven to be an excellent metabonomic tool and applied in metabolites identification and quantification based on its convenience, high sensitivity, peak resolution, and reproducibility. In this study, we used IC to detect urine-free amino acids profiles of early gastric cancer, advanced gastric cancer, and health people. Amino acids in the human body undergo interdependent regulation; comparing single amino acid concentration between patients and controls might be insufficient to elucidate amino acid changes associated with cancer development. Therefore, differences in amino acid profiles from the three groups were characterized by principal components analysis (PCA) in the present study. Based on pattern results, we tried to construct a diagnostic model to discriminate gastric cancer from healthy individuals and another diagnosis model for clinical staging.

## 2. Materials and Methods

### 2.1. Materials

All standards and samples were prepared with deionized water (Labconco, Kansas City, MO, USA). Sodium hydroxide (NaOH) (50%, w/w) was purchased from Fisher Scientific (Hampton, NH, USA). Omithine (≥99.5%), cystine (≥99%), sodium acetate (≥98%), and amino acid standard solutions were purchased from Sigma-Aldrich (St. Louis, MO, USA). Spermidine trihydrochloride (>98%) and spermine tetrahydrochloride (≥99%) were purchased from Calbiochem (San Diego, CA, USA).

### 2.2. Sample Collection and Preservation

Twenty six in-patients, aged 53 to 86 years and diagnosed with gastric cancer, were categorized according to endoscopic examination coupled with histopathological features and stages according to the seventh edition of the International Union Against Cancer (UICC) TNM: stages I and II  (early-stage cancer), 7 patients (female/male, 3/4), aged 53 to 86 years (the median age was 72 years old); stages III and IV  (advanced-stage cancer), 19 patients (female/male, 9/10), aged 54 to 84  years (the median age was 76 years old). Patients enrolled in this research were not on any medication before sample collection. The clinical diagnosis and pathological reports of all the patients were obtained from the hospital. Seventeen healthy subjects (female/male,  8/9), aged 50 to 86 years (the median age was 68 years old), were selected by a routine physical examination including endoscopy, and any subjects with chemotherapy, kidney disease, and endocrine disorders were excluded. Urine samples were collected in the morning before breakfast from a total of 26 gastric cancer patients and 17 healthy volunteers at Shanghai Sixth Hospital, Medical College of Shanghai Jiao Tong University (Shanghai, China). All the patients and subjects were Han Chinese living in China and had normal nutritional status. The protocol was approved by the Shanghai Sixth Hospital Institutional Review Board, and all participants gave informed consent before they were involved in the study. In this study, we used IC to detect urine free amino acids profiles of gastric cancer and health of people. To study urine-free amino acids profiles for screening or diagnosing gastric cancer  especially for  early gastric cancer, urine samples were divided into 3 groups.

### 2.3. Ion Chromatography

The chromatography system consisted of a Dionex ICS-3000 Reagent-Free TM Ion Chromatograph (Dionex Corporation, Sunnyvale, CA, USA) with a DP-3000 dual gradient pump, a DC-3000 detector compartment with a conductivity cell and an electrochemical cell, an EG-3000 eluent generator with an EluGen EGC II MSA cartridge, and an AS autosampler.

Amino acids were separated with an AminoPac PA10Pac CS18 (250 mm × 2 mm I.D., Dionex Corporation) analytical column and its respective guard column, CG18 (50 mm × 2 mm I.D.) with a flow rate of 0.25 mL/min and a thermostated temperature of 30°C. A CSRS ULTRA II (2 mm) self-regenerating suppressor operating at 40 mA in the external water mode was used for suppressed conductivity detection. A 25 *μ*L sample injection volume was used throughout the experiment. Operating backpressure was less than 3,000 psi. The gradient elution conditions consisted of deionized water from 0 to 42 min, 40 mM to 200 mM NaOH from 0 to 42 min, and 400 mM to 700 mM sodium acetate from 18 to 42 min.

### 2.4. Statistical Analysis

After the chromatographic peak area was normalized, the PCA analysis was done to construct urine amino acid metabolic profile of different stages of gastric cancer patients and control subjects. All data were expressed as mean ± SD. Statistical analysis was performed using Wilcoxon rank sum test. *P* < 0.05 was considered statistically significant.

## 3. Results

### 3.1. Chromatogram of 22 Amino Acid Standard Solution

**Figure 1:**
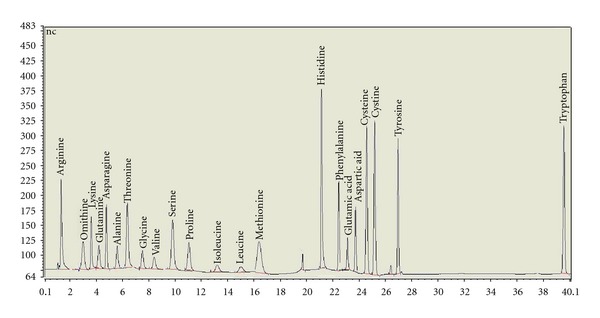
IC chromatogram of 22 amino acids standard solution: (1) arginine; (2) omithine; (3) Lysine; (4) glutamine; (5) asparagine; (6) alanine; (7) threonine; (8) glycine; (9) valine; (10) serine; (11) proline; (12) isoleucine; (13) leucine; (14) methionine; (15) histidine; (16) phenylalanine; (17) glutamic acid; (18) aspartic aid; (19) cysteine; (20) cystine; (21) tyrosine; (22) tryptophan.

From Figure 1, it was showed that 22 amino acids were all separated effectively in 40 minutes. The concentration of standard solution was 8 *μ*M.

### 3.2. Total Ion Current Chromatogram for Health Adult Group and Gastric Cancer Group

**Figure 2:**
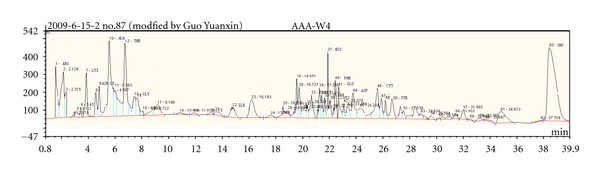
Total ion current of a normal subject urine.

**Figure 3:**
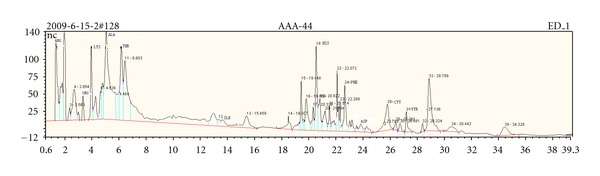
Total ion current of a gastric cancer inpatient urine.

As can be seen from Figures 2 and 3, the urine chromatograms of health adult group and gastric cancer group detected by IC showed that the total ion current (TIC) peaks of two groups were different. At the same retention time, peak size and peak height were different between groups.

After IC analysis, each sample was represented by a TIC, and the peak areas of amino acids were integrated. We qualitatively analyzed 22 amino acid chromatogram of each urine sample according to the chromatogram of 22 amino acid standard solution, and the peak-area ratio of each compound to a corresponding internal standard was calculated as the response by using peaknet6 software. Statistical analysis was performed using Wilcoxon rank sum test.  Table 1 showed that the urine-free amino acid profiles of gastric cancer changed to a certain degree compared with healthy adult subjects. Compared with healthy adult group, the levels of valine, isoleucine, and leucine increased (*P* < 0.05), but histidine and methionine decreased (*P* < 0.05), and aspartate decreased significantly (*P* < 0.01) in gastric cancer patients.

When we compared urine amino acid profiles of early gastric cancer to advanced gastric cancer, valine level decreased (*P* < 0.05) and isoleucine level remarkably decreased in advanced gastric cancer (*P* < 0.01) as shown in Table 2.

### 3.3. Pattern Recognition

**Figure 4:**
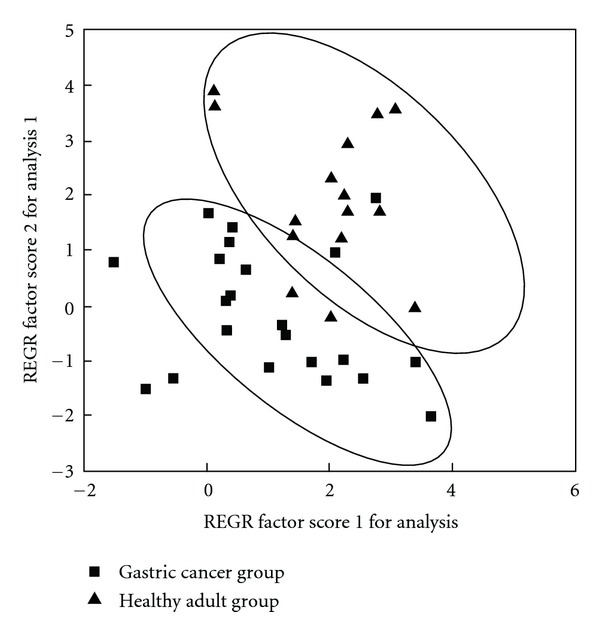
PCA scores plot of urine-free amino acids in healthy adult group and gastric cancer group (diagnosis model for gastric cancer).

**Figure 5:**
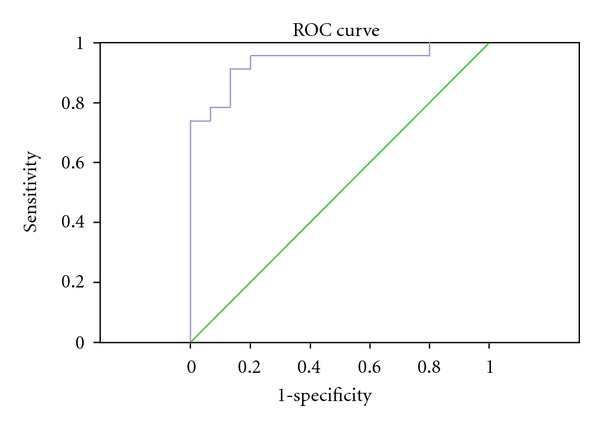
ROC of diagnosis model for gastric cancer (AUC = 0.936).

SPSS16.0 software is used for PCA analysis of the data; PCA scores plot showed that different urine samples (healthy control and gastric cancer groups) were scattered into two different regions (Figure 4). ROC analysis, which was performed using the values determined by the first two components of the PCA model, confirmed the robustness of the PCA model. The sensitivity and specificity trade-offs were summarized for each variable with the area under the curve (AUC). The AUC value of this PCA model was 0.936 (Figure 5), which demonstrated a good differential value for gastric cancer.

**Figure 6:**
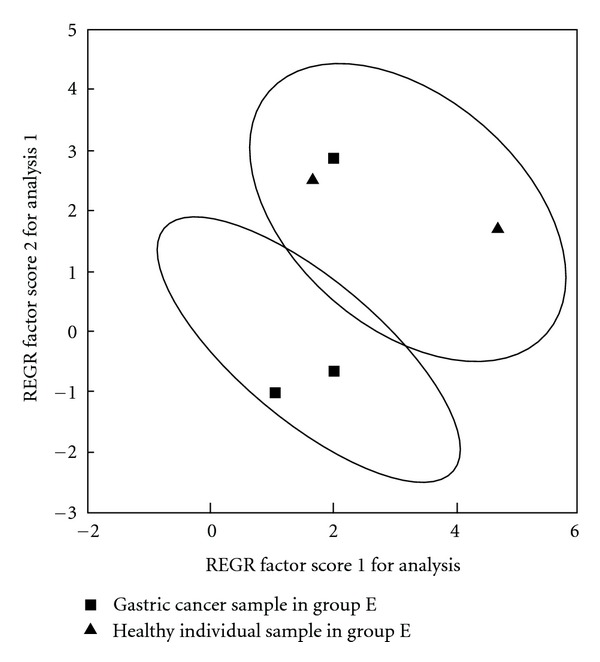
PCA of urine-free amino acids in group E (test result of diagnosis model for gastric cancer).

We make PCA according to PC1 and PC2 of five tested samples to test the diagnosis model for gastric cancer (Figure 6), and we can see that two cases of normal samples are all in the normal region, and that two cases of gastric cancer samples are in the cancer region except one case of gastric cancer is in the noncancer region.

**Figure 7:**
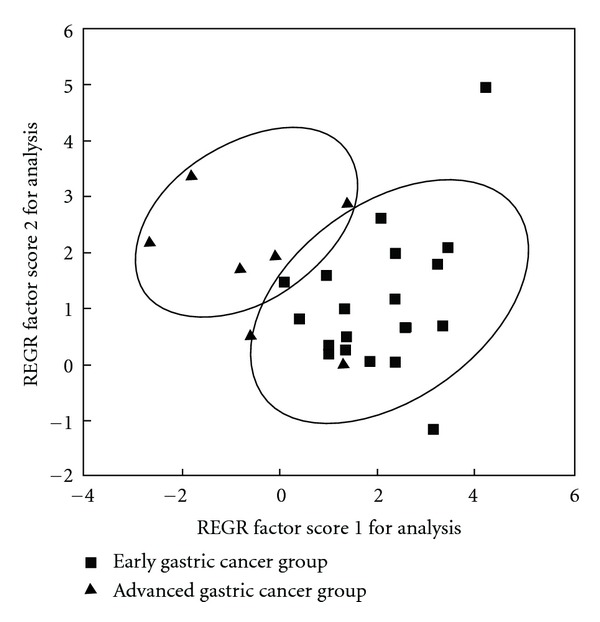
PCA of urine free amino acids in early and advanced gastric cancer group (diagnosis model for clinical staging).

**Figure 8:**
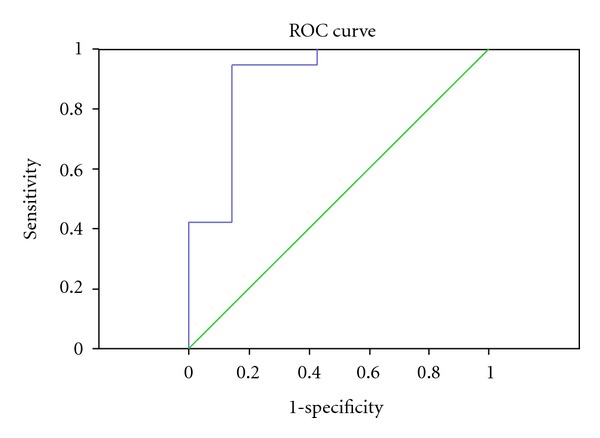
ROC of diagnosis model for clinical staging (AUC = 0.902).

PCA was also performed to differentiate between early and advanced gastric cancer groups.  Figure 7 showed that most urine samples from early gastric cancer were separated from advanced cancer samples. This PCA model was also validated by ROC analysis (AUC = 0.902, Figure 8).

**Figure 9:**
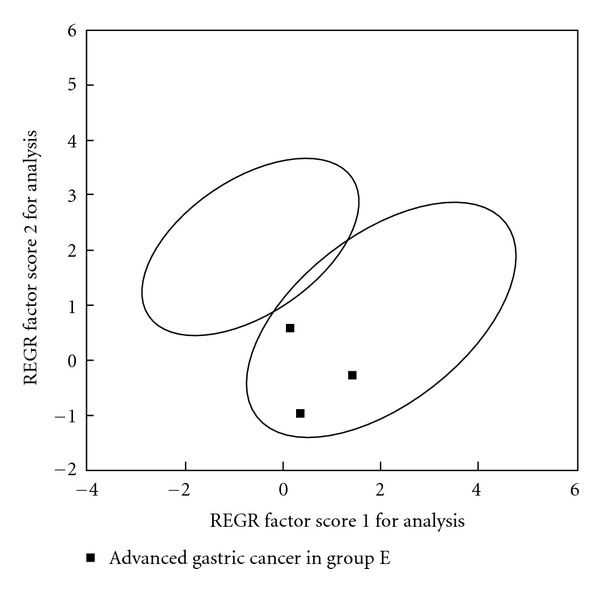
PCA of urine free amino acids of 3 advanced gastric cancer in group E (test result of diagnosis model for clinical staging).

There were no early gastric cancer samples in tested samples, so we just take three cases of advanced gastric cancer samples to PCA (Figure 9), and we can see that 3 cases of advanced gastric cancer samples are all in the advanced cancer region.

## 4. Discussion

 In the current study, we performed urine amino acid profile to identify marker metabolites. Some amino acids were differentially expressed in patients with gastric cancer and control subjects. Diagnosis model for gastric cancer which was tested by a small-scale sample showed its potential value in clinical diagnosis. The high AUC value indicated that the PCA model was robust in the discrimination. Another diagnosis model for gastric cancer staging which was also tested by a small-scale sample was of potential value in clinical diagnosis. Figure 7 showed that some of samples from early gastric cancer were located at advanced gastric cancer samples. Cancer can progress quantitatively or qualitatively, and these patients may be in the intermediate stage from early gastric cancer to advanced gastric cancer.

It has been reported that amino metabolism is remarkably perturbed in cancer cells [4, 33], and urine amino acid profiles are also altered [12, 34–36]. Changes in amino acid metabolism and an increase in gluconeogenesis have been well documented in cancer patients [35, 36]. In the present study, the model identified patients at early stage of gastric cancer and advanced gastric cancer, suggesting that the urine amino acid profiling is useful for diagnosis of gastric cancer.

The results showed that the isoleucine, leucine, and valine levels in urine of patients with gastric cancer were significantly higher than those in normal controls. As malignant tumors grow rapidly, they need a large number of amino acids from the metabolism pool as a substrate for synthesis of proteins and nucleic acids and other substances. The metabolism of amino acids in body's muscle tissue is above 50% of the total metabolism, and the catabolism of branched-chain amino acids (BCAA), such as valine, leucine, and isoleucine, is mainly involved in skeletal muscle. So tumor tissues have high demand on the BCAA. Many experiments showed that patients with malignant tumors, including gastric cancer, tended to be high metabolic. Isoleucine is a glucogenic and ketogenic amino acid which decomposed into acetyl-coenzyme A and succinate-coenzyme A, the important materials in the citric acid cycle, which were greatly required during the gluconeogenesis [19], so the level of isoleucine in the gastric cancer patients is higher than the normal group. Most cancer cells predominantly used amino acids to produce more energy by glycolysis but not oxidative phosphorylation via the tricarboxylic acid (TCA) cycle [33, 37]. Valine is a glucogenic amino acid, and leucine is a ketogenic amino acid. So, the levels of isoleucine, leucine, and valine which are important to the process of gluconeogenesis in the cancer patients are higher. As tumor cells have an active nucleic acid metabolism, histidine was substantially absorbted into tumor tissue, and consumption significantly increased, leading to its decline in body. Moreover, the metabolism of specific amino acids is known to be related to specific organs, such as muscle, or liver, and changes in the levels of amino acids are affected by their metabolism in organs of the body. Some amino acids were reported to correlate with specific cancers [17, 38]. Therefore, profiling plasma or urine amino acids can detect the metabolic alterations in specific organs, which may be applied in early cancer diagnosis.

 From the present study, the isoleucine and valine levels in urine of patients with early gastric cancer were slightly higher than those of advanced gastric cancer. It may be because protein was excessively consumed in advanced cancer, which leads to a lower level of valine than in early cancer group. Yamanaka et al. made a hypothesis: the increased obstacles in muscle tissue protein improved the glucose gluconeogenesis in hepatic and increased the oxidation of BCAA in muscle, which was the main mechanism for the rise of BCAA levels in the patients of early gastric cancer; tumor can stop the further development of this increased muscle protein barrier, and then intravenous BCAA and EAA levels may decline.

In conclusion, MS-based techniques, such as liquid chromatography/mass spectrometry (LC/MS) including ion chromatography and gas chromatography/mass spectrometry (GC/MS), are very important tools in the diagnosis of many diseases. In this study, we found that amino acid balance in stomach cancer patients was significantly different from the healthy individuals, also there were differences between early gastric cancer and advanced gastric cancer. We believe that ion chromatography technique has great potential in the early diagnosis or screening of diseases especially gastric cancer and is worthy of further evaluation and research.

## Figures and Tables

**Table 1:** Urine amino acid profiles of healthy adult group and gastric cancer group [*R* = (healthy adult group − gastric cancer group)/gastric cancer group].

Amino acids	Healthy group (group A)	Gastric cancer (group B + C)	*R*	*P*
Isoleucine	1.640 ± 0.771	2.539 ± 1.661	−0.354	0.024
Leucine	2.289 ± 1.162	3.426 ± 2.376	−0.332	0.047
Valine	0.789 ± 0.863	1.535 ± 1.128	−0.486	0.033
Methionine	3.551 ± 1.931	1.997 ± 1.249	0.778	0.011
Histidine	3.292 ± 1.885	1.882 ± 0.837	0.749	0.014
Aspartic aid	3.312 ± 1.594	1.620 ± 1.468	1.044	0.001

**Table 2:** Urine amino acid profiles of early and advanced gastric cancer groups [*R* = (early gastric cancer group − advanced gastric cancer group)/advanced gastric cancer group].

Amino acids	Early gastric cancer group	Advanced gastric cancer group	*R*	*P*
Isoleucine	3.946 ± 1.982	2.023 ± 1.214	0.951	0.006
Valine	2.568 ± 1.506	1.155 ± 0.665	1.223	0.048
